# Predicting the behavioural tendency of loss aversion

**DOI:** 10.1038/s41598-019-41242-w

**Published:** 2019-03-22

**Authors:** Jianmin Zeng, Yujiao Wang, Jing Zeng, Zhipeng Cao, Hong Chen, Yijun Liu, Qinglin Zhang, Li Su

**Affiliations:** 1 Sino-Britain Centre for Cognition and Ageing Research, Faculty of Psychology, Southwest University, Chongqing, China; 2 Department of Education Science, Liupanshui Normal University, Liupanshui, Guizhou China; 3 International college, Krirk University, Bangkok, Thailand; 4 Department of Psychiatry, University of Cambridge, Cambridge, UK

## Abstract

Loss aversion manifests itself in rejecting a gamble of gaining or losing the same amount of money with equal chance. Although loss aversion is a well-known and intensively studied phenomenon, whether individual differences in behavioural loss aversion can be predicted using scalp potentials (EEG) remains unclear. The current study measured scalp potential when subjects played a series of fair coin-toss games in three situations (high, medium and low loss conditions). We found that most people chose to bet in the low loss condition and avoided to bet in the high loss condition. However, their betting behaviour in the medium loss condition was variable, reflecting their different behavioural tendency of loss aversion. We found that late positive complex (LPC) significantly increased when subjects accepted gambles in the low loss condition (implying potential loss), relative to when they rejected gambles in the high loss condition (implying no gain and no loss), which is consistent with the previous finding that emotional stimuli can induce enhanced LPC. We further found that the difference in the scalp potentials from the above two conditions could predict behavioural tendency of loss aversion in the medium loss condition across subjects. This result demonstrated how behavioural tendency in a situation can be predicted using EEG from other situations.

## Introduction

Are you willing to accept a gamble in which you have a 50 percent chance of gaining $100 and an equal chance of losing $100? If you are loss-averse, then you will reject this gamble.

In prospect theory^1,2^, Kahneman and Tversky proposed that human beings are more sensitive to losses than they are to comparable gains when making decisions. They modelled loss aversion by a value function or utility function with an asymmetric “S” curve. In their model, losses and gains are defined relative to a certain neutral reference point (usually the status quo), and the slope of the utility function for losses is steeper than that for gains, reflecting the fact that humans are more sensitive to losses. According to previous studies^2^, for a gamble with 50% chance to get $X and 50% chance to lose $Y, an average person is willing to accept this gamble when X ≥ 2Y. Nevertheless, some scholars^3^ argued that loss aversion is typically observed for large but not small outcomes and this case is inconsistent with the original suggestion^1^ by Kanheman and Tversky that loss aversion is general.

As one of the basic principles in risky and riskless decision-making^2,4,5^, loss aversion explains several well-known economic phenomena. (1) The endowment effect: The minimal acceptable price for selling you-owned goods is generally higher than the maximum acceptable price for buying the same goods from others. That is because when you sell goods, you lose goods and get money, and you are more sensitive to losing goods; when you buy goods, you lose money and get goods, and you are more sensitive to losing money^4,6,7^. (2) The status quo bias: Individuals have a strong preference for the current state rather than changing it. It is because the disadvantages (loses) of changing the current state often loom larger than the advantages (gains) of changing it^8–10^.

In the last 30 years, numerous studies^11–25^ have investigated loss aversion. Some scholars found that people typically overestimate the future emotional impact of losses^26^. Psychophysiological studies have also shown that the skin conductance response (SCR) to losses was significantly greater than the SCR to equivalent gains^27^. Pupil dilation and heart rate^28^ in gain and loss conditions also exhibited similar differences, suggesting humans are really more sensitive to loss than to gain. 

Neuroimaging study using functional magnetic resonance imaging (fMRI) demonstrated that loss was associated with the de-activation of a network of brain regions, including the striatum, the ventromedial prefrontal cortex (VMPFC), the ventral anterior cingulate cortex (ACC), and the medial orbitofrontal cortex (OFC)^11^. More critically, a gain/loss conjunction analysis revealed that key regions in the striatum and VMPFC exhibited a pattern of “neural loss aversion”: The decreased activation of these regions in response to losses was greater than the increased activation in response to gains for most participants. Nevertheless, some scholars^3^ argued that in that study, the losses were typically smaller than the gains, and all brain signals were normalized for reward magnitude; thus, diminishing sensitivity of the value function^1,2^ could be an alternative explanation for the observed results. The results also demonstrated that the brain activation in several regions, including the bilateral ventral striatum, significantly correlated with the participants’ behavioural loss aversion^11^. In addition, asymmetric BOLD responses to positive and negative outcomes have also been found in^29^.

Many studies have provided valuable insights into the cognitive mechanisms and neural correlates of loss aversion, but to our knowledge, few studies have studied individual differences in behavioural loss aversion (IDBLA) using electrophysiology, which is a more direct measure of neural activity than fMRI. The current study used event-related potential (ERP) to investigate whether IDBLA can be predicted using ERP.

In this study, participants made a series of decisions to accept or reject a fair coin-toss gamble, in which the probability of winning and losing were both 50% throughout the experiment. However, we set the ratio between the potential losses and gains at three different levels. (1) *High Loss Condition*, where the losses were approximately equal to the gains. In this condition, people would generally reject the bet according to previous research^2^. For this condition, we would analyse ERPs of rejecting bets but not accepting bets because the trial number of accepting bets would be too small for a within-subject ERP average. (2) *Medium Loss Condition*, where the losses were approximately half of the gains. In this condition, participants as a whole would accept or reject the gamble with approximately equal probability according to previous research^2^, but individuals would exhibit a spectrum of IDBLA. For this condition, we would analyse IDBLA. We would not analyse ERPs of betting or rejecting, because for some subjects the trial number of betting or rejecting would be too small for a within-subject ERP average. (3) *Low Loss Condition*, where the losses were 1/10 to 1/5 of the gains. In this condition, most people would choose to bet according to previous research^2^. For this condition, we would analyse the ERPs of betting but not rejecting because the trial number of rejecting would be too small for a within-subject ERP average.

For ERP analysis, given that the decision task in this study was a higher-level mental activity, we focused on the late positive component (LPC). It is well-established that enhanced LPC is associated with emotional stimuli compared with neutral or less emotional stimuli. This effect is robust for different stimulus types such as object pictures^30^, human pictures^31,32^ and words^33–35^. In our study, rejecting bets in the high loss condition would always result in no gain and no loss, i.e. remaining in the status quo, and thus can be seen as a neutral or baseline reference condition. In contrast, accepting bets in low loss condition, although the loss was small, could in theory result in a loss at half of the times; so, compared with rejecting the bets in the high loss condition, one would experience stronger emotion (e.g., more worry about the potential losses) when accepting bets in the low loss condition. Based on previous findings, we hypothesized that relative to rejecting bets in the high loss condition, accepting bets in the low loss condition would result in an enhanced LPC.

Therefore, individuals who were more sensitive to losses, would experience stronger emotion when betting in low loss condition (when possibly losing something) than when not betting in high loss condition (when remaining in the status quo), and thus would have larger LPC difference between the two conditions. Therefore, we can take the LPC difference as a neural index of loss sensitivity. Given that loss aversion is rooted in loss sensitivity^1,2^, more loss-sensitive individuals (with larger LPC difference), should have stronger behavioural tendency of loss aversion, which could be measured in medium loss condition. Therefore, we further hypothesized that the LPC difference between betting in low loss condition and not betting in high loss condition could across subjects predict the behavioural tendency of loss aversion in medium loss condition.

## Results

### Behavioural results

Only 11 out of the 7200 trials (20 subjects * 360 trials/subject) received no response. Consistent with previous studies, the proportion of trials in which subjects chose to gamble was 12% in the high loss condition, 57% in the medium loss condition, and 84% in the low loss condition. Furthermore, most subjects chose not to bet in most trials in the high loss condition (“most” is defined as at least 75% in this paragraph). Most subjects chose to bet in most trials in the low loss condition. As expected, the individuals’ proportions of betting in the medium loss condition were variable.

Furthermore, we tested correlations between IDBLAs in all the three trial types (low, medium and high) using Spearman’s ranked correlation. As shown in Table 1, IDBLAs of three conditions were significantly correlated with each other, suggesting that there was a common psychological process underlying all three conditions.

**Table 1:** Correlations among IDBLAs of three conditions.

Statistical method	Correlation Coefficient			*P* value (2-tailed)		
	hig-low	hig-med	low-med	hig-low	hig-med	low-med
Spearman’s *rho*	0.7	0.735	0.876	0.001	0.0002	0.0000004

Note. hig, low, and med denote high, low, and medium loss condition respectively.

We also analysed the response times. Please see the Supplementary Information for the details.

### Electrophysiological results

**Figure 1:**
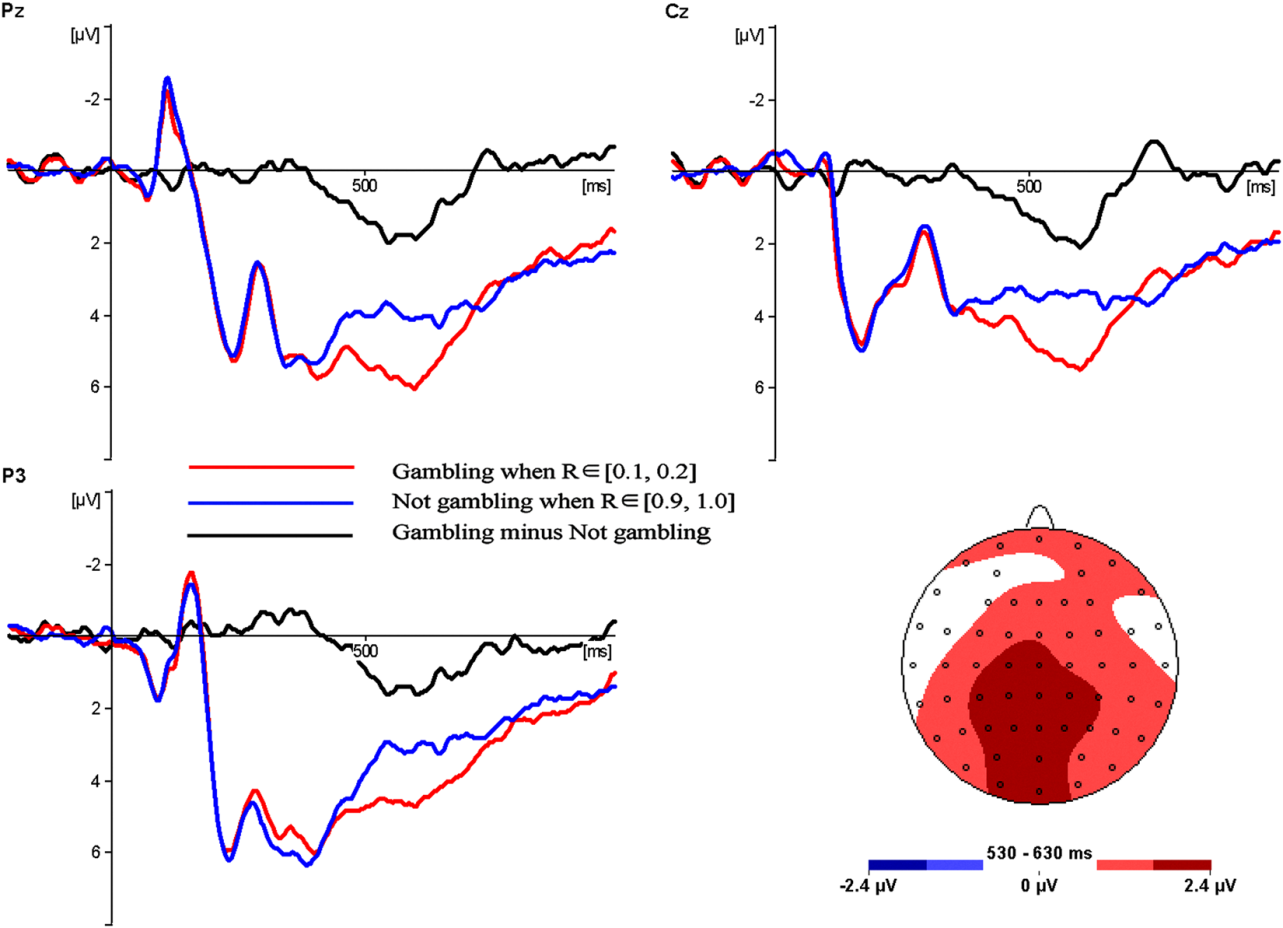
Waveforms and topography.

The following 14 electrode sites were selected for statistical analysis within the LPC (530–630 ms) time window: C1, Cz, CP1, CP2, CP3, CPz, P1, P2, P3, Pz, PO3, POz, O1, and Oz (See lower right panel of Fig. 1). The EEG time series were time-locked to the onset of the stimuli. The average amplitude of this time window was analysed using repeated measures analysis of variance (ANOVA), with independent variables being the decision type (gambling in the low loss condition versus not gambling in the high loss condition) and the electrode site. The P-values of the analysis of variance were corrected using the Greenhouse-Geisser correction.

The main effect of the decision type was significant (*F* (1, 19) = 4.473, *p* = 0.048, η^2^_*p*_ = 0.191). The main effect of the electrode site was also significant (*F* (13, 247) = 7.622, *p* = 0.001, η^2^_*p*_ = 0.286). However, the interaction between the decision type and the electrode site was not significant (*F* (13, 247) = 0.321, *p* = 0.833, η^2^_*p*_ = 0.017). Actually, LPC related to gambling (M = 4.47 μV, SE = 1.09 μV) were significantly larger than those related to not gambling (M = 2.71 μV, SE = 1.08 μV) during the 530–630 ms time window (Fig. 1).

### Combined analysis of behavioural and electrophysiological data

As previously discussed, we wanted to explore the relation between IDBLA in the medium loss condition and LPC differences between the high and low loss conditions. For this purpose, we calculated two indices for each subject. First, the behavioural index of loss aversion in the medium loss condition. It is the difference between the percentage of trials in which the subject rejected the gamble in the medium loss condition and the percentage of trials in which the subject accepted the gamble in the medium loss condition. A more positive value indicates more loss aversion in behaviour. Second, the scalp potential index, which was calculated as the difference between the average LPC of the trials in which the subject accepted the gamble in the low loss condition and the average LPC of the trials in which the subject rejected the gamble in the high loss condition. As previously discussed, a more positive value would indicate more worry about a betting-related potential loss.

**Figure 2:**
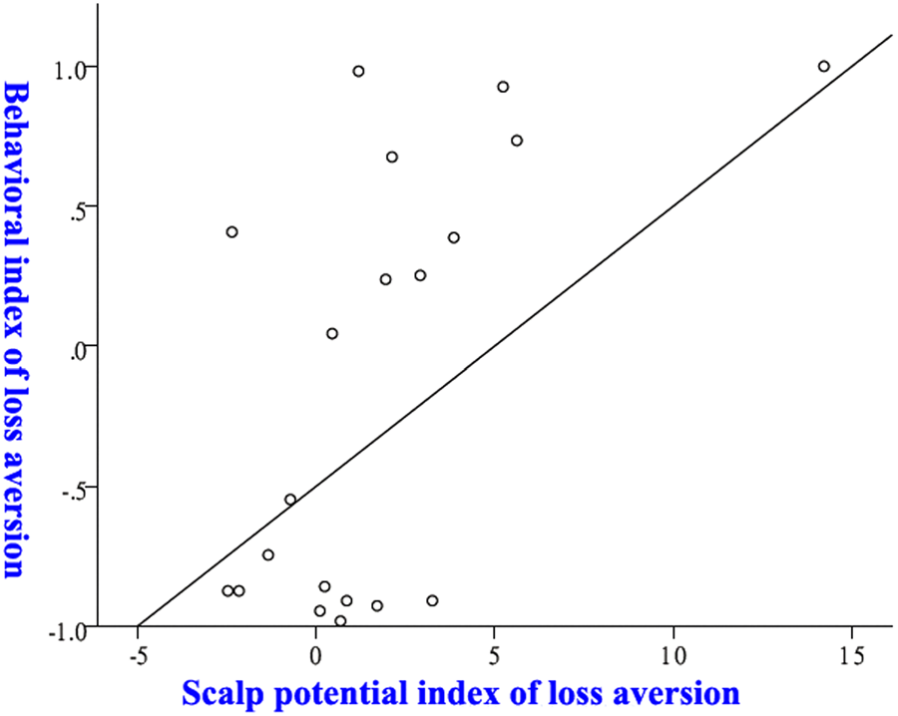
Predicting IDBLA in one situation using scalp potentials from other situations.

Linear regression and Pearson’s correlation analysis revealed that scalp potential index predicted and significantly correlated with the behavioural index of loss aversion across all subjects (*p* = 0.007, r = 0.581, n = 20). Figure 2 depicts the correlation and prediction between these indices. This result remained significant, even after removing a possible outliner - the rightmost data point (Fig. 2) from the analysis (*p* = 0.020, r = 0.529, n = 19). These results indicated that IDBLA showed in the medium loss condition could be predicted using characteristic scalp potentials profile from other conditions.

## Discussion

Loss aversion refers to the tendency of rejecting a gamble with an equal chance to win or lose the same amount of money. This phenomenon can be explained as the emotional impact of losses is larger than that of the equal amount of gains, and can be modelled using a subjective value function from the prospect theory, in which the slope is steeper for loss than it is for gain. Many researchers have used loss aversion as an explanation for a wide range of economic phenomena. The current study used ERPs to investigate the neural correlates of loss aversion when subjects made decisions to accept or reject a series of mixed gambles.

Our behavioural results showed that subjects chose to bet in most trials in the low loss condition, avoided betting in most trials in the high loss condition, and gambled in approximately half of the trials in the medium loss condition. As expected, these behavioural results are consistent with previous findings^2^.

We found that accepting the gamble in the low loss condition elicited a larger ERP in the 530–630 ms time window than rejecting the gamble in the high loss condition. This component should be the late positive complex (LPC) based on the ERP waveform and topographical distribution (see Fig. 1).

As mentioned in Introduction, LPC is often associated with emotion processing^30–35^. In the current study, betting in the low loss condition (despite the potential loss being relatively small) would trigger a stronger negative emotion (such as worry, anxiety, or fear) than not betting in the high loss condition (a reference condition representing the status quo) and thus induced a larger LPC.

Larger LPC amplitudes have also been argued to reflect that more resources were deployed for the underlying mental processing^36,37^. Making a decision to accept the gamble required more extensive evaluation and analysis of potential loss and gain, and was associated with stronger emotional burden. Moreover, subjects who accepted gambles would continue to engage with the task for the potential outcomes. This would require more cognitive resources and emotional processing, and thus induced a more positive LPC than decisions to reject the bet (which effectively disengaged the relevant mental processes).

As the LPC time window partially overlaps with several other well-known ERP components, such as P300 and N400, the current results could in theory be re-interpreted by difference in risk levels. The P300 amplitudes were larger in high-risk than in low-risk choices^38^ and a greater amplitude of N400 has also been found to be related to increased risks^39^. However, this interpretation was inconsistent with our findings that the LPC was greater in the low loss condition, in which the overall risk was the lowest among all three conditions.

Another alternative explanation of the LPC results is response-related ERP. Specifically, the action related to betting might induce a negative response-locked ERP than not betting^40^. However, our EEG time series were time locked to stimulus onset rather than response. As the reaction time varied between subjects and between trials, the LPC is unlikely to reflect merely the action of the decision itself. So, it is unlikely that our data would support this alternative explanation.

Most importantly, we found that IDBLA in the medium loss condition could be predicted using the difference in LPC between high and low loss conditions. Subtracting the LPC of trials in which the subjects chose not to bet in the high loss condition from the LPC of trials in which the subjects chose to bet in the low loss condition revealed a neural index indicating the subjects’ sensitivity to the potential loss. This neural sensitivity to potential loss could therefore predict the subjects’ behavioural tendency of loss aversion in the medium loss condition. We argue that this mechanism explains why individual differences in behavioural tendency of loss aversion, can be predicted using LPC.

Compared with previous research using fMRI data to predict behavioural loss aversion across subjects^11^, our study complements it by providing additional temporal information on when the loss-aversion bias starts to have an effect. This has implications about whether it is an early stimulus driven or later higher order cognitive bias. Our findings support that although loss aversion can be implicit and often without overt control, it is at a relatively late stage of decision-making.

In conclusion, the subjects in the present experiment participated in a fair coin-toss game, and their scalp potentials were processed using ERP technique. Electrophysiological scalp data revealed that betting in the low loss condition was associated with larger LPC than not betting in the high loss condition. The difference of LPC amplitude might reflect individual sensitivity to potential losses, and thus could predict behavioural tendency of loss aversion across subjects.

## Methods

### Participants

Twenty undergraduates (10 females; average age being 23.5 years) volunteered to participate in the experiment. Students who majored in Psychology, Mathematics and Statistics, or Economics and Management were excluded because we wanted the responses of normal subjects without relevant academic background. The subjects were all right-handed, with normal or corrected-to-normal vision and no history of mental illness. All subjects signed written informed consent prior to participation and received a certain amount of money according to his or her decision-making results. The Administrative Committee of Psychological Research at Southwest University approved this study, and the experiment was performed in accordance with the approved guidelines.

### Experimental procedure

**Figure 3:**
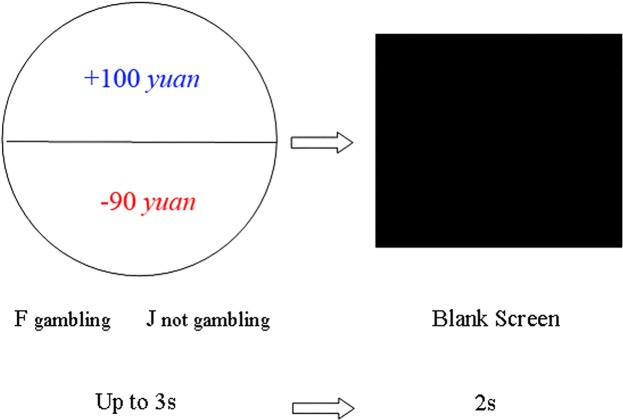
Illustration of a typical trial. First, a circle with +X *yuan* and −Y *yuan* in the upper and lower halves displayed at the centre of the screen until a subject decided to bet or not by pressing a corresponding key within 3 s. Second, a blank screen was presented for 2 s.

A participant’s task in a typical trial of this experiment was to decide whether to participate in a fair coin-toss gamble. In the gamble, if the head of coin faced up, then the subject would receive X *yuan*, but the subject would lose Y *yuan* if the tail faced up. The subject had an equal chance (50/50 chance) of gaining X *yuan* or losing Y *yuan*. The potential gain (+X *yuan*, coloured in blue) and loss (−Y *yuan*, coloured in red) were simultaneously presented in the upper and lower halves of a circle displayed in the centre of the screen. As exemplified in Fig. 3, if the participant chose to gamble, then he/she would gain 100 *yuan* or lose 90 *yuan*, depending on which side of the coin faced up. If the participant chose not to gamble, then he/she would neither gain nor lose any money.

The value of potential gains (X) ranged from 100 to 990 yuan, and the value of potential losses (Y) was proportional to X, i.e., Y = LX, with 0.1 ≤ L ≤ 0.2 for the low loss condition, 0.5 ≤ L ≤ 0.6 for the medium loss condition, and 0.9 ≤ L ≤ 1.0 for the high loss condition. These ranges were selected according to a previous study^2^, which suggested that the average person is roughly indifferent between ($2X, 50%; $-X, 50%) and ($0, 100%). Therefore, the subjects as a whole in the present study would feel roughly indifferent between betting and not betting in the medium loss condition, but would likely exhibit a spectrum of individual differences. Subjects would overwhelmingly choose not to bet in the high loss condition and would overwhelmingly choose to bet in the low loss condition.

The subjects were asked to choose to bet or not bet by pressing a corresponding key within 3 seconds. However, the gamble was not resolved during collection of scalp potentials, which implied that the subject did not know the gambling result after the decision was made. This procedure ensured that the cognitive and affective processes of a trial were not contaminated by the outcomes of the last trial. After the subject’s response of betting or not, a blank screen was subsequently presented for 2 seconds before the next trial.

Each participant first completed 18 practice trials, which were divided into 3 stages. The decision time was not limited in the 1^st^ stage, and the participant could take his/her time to make the decision. The decision time was limited to 3 seconds in the 2^nd^ stage. The 3^rd^ stage was the same as the 2^nd^ stage, except the subject was told that the trials were formal trials (although the trials were actually still practice trials) so as to make a smooth transition from practice to fomal tests. Practice trials were not marked nor analysed.

Each participant subsequently completed 360 trials in the formal experiment, which were divided into 4 sessions of 90 trials. The participant took breaks before the 2^nd^, 3^rd^ and 4^th^ sessions. The first 10 trials in the 2^nd^, 3^rd^, and 4^th^ sessions were treated as practice so as to exclude the possible influence of breaks, and these trials were not marked nor analyzed. All trials were presented in a random order for each subject. The corresponding response keys were counter-balanced within and between subjects: Subjects with odd numbers pressed “F” for gambling and “J” for not gambling in the 1^st^ and 3^rd^ sessions, and pressed “F” for not gambling and “J” for gambling in the 2^nd^ and 4^th^ sessions. Even-number subjects had the opposite arrangement.

Each participant wore an electrode cap and was seated in a quiet room. The participants’ eyes were approximately 80 cm away from the computer screen, with maximum visual angles for the stimuli being 8.2° (horizontal) × 3.9° (vertical). All stimuli were presented in the centre of the screen. The participants were required to fixate their eyes on the centre of the screen during the experiment and minimize blinking and body movements while performing the tasks. The subjects were instructed to press “F” with their left index finger and “J” with their right index finger to indicate their choices.

We instructed each subject to make his decision based on instincts, and after the experiment, he would receive a compensation consisting of two parts: a basic payoff of 10 yuan and a performance based payoff as explained below. After he finished the experiment, three decision questions would be randomly chosen from all decision questions. For each of the 3 questions, if he had chosen not to bet, then the decision outcome would be 0 yuan; but if he had chosen to bet, then the decision outcome would be determined by tossing a coin. If the head (tail) faced up, then the decision outcome would be the gain (loss) in the question. The 3 decision outcomes would then be summed, and the results were linearly transformed into a decision payoff. After completing the experiment, the subject was paid according to the above instructed payoff mechanism, with the decision payoff falling into the range of −2 to 10 yuan.

### Electrophysiological recording and analysis

The ERP recording and analysis system was manufactured by the Brain Products Company (Munich, Germany). This system uses an elastic cap with extended electrodes at 64 scalp sites in accordance with the international 10–20 system to record the electrical activity on the participants’ scalps. The reference point was placed on the left mastoid. One electrode was placed on the outside rim of each eye to record the horizontal electrooculogram (HEOG) generated from horizontal eye movement. Another two electrodes were placed approximately 1 cm above and below the right eye to record the vertical electrooculogram (VEOG) generated from blinks and vertical eye movements. All electrode impedances were maintained below 10 kΩ. The EEG and EOG signals were amplified and digitized using a sampling frequency of 500 Hz and a filter bandpass of 0.1–100 Hz.

The EEG data were subsequently preprocessed offline. The data were first re-referred to the average amplitude of left and right mastoids. Eye movement artefacts (eye blinks and movements) were corrected using the Gratton & Coles method. The EEGs were filtered with a high cutoff of 16 Hz, 12 dB/oct, segmented and baseline corrected. Segments whose peak voltages exceeded ±80 μV after correction were excluded before averaging. At least 33 segments were retained for each electrode in each ERP condition under consideration for each subject, except one subject’s one condition had only 14 segments. The inclusion or exclusion of this subject’s data produced similar results, and the final report was based on the data from all twenty subjects. These steps were performed using Brain Vision Analyzer software (Brain Products). The ERP segments were time locked to the onset of the decision-making screen. The averaged epochs for analysis included a 1000 ms post-stimuli waveform and a 200 ms pre-stimuli baseline.

## Supplementary information

Supplementary Information
