# Supplementary material for: Predicting the behavioural tendency of loss aversion
Source: Sci Rep. 2019 Mar 22;9:5024. doi: 10.1038/s41598-019-41242-w (PMC6430803; doi:10.1038/s41598-019-41242-w)

***Supplementary Information to***

**Predicting the Behavioural Tendency of Loss Aversion**

Jianmin Zeng^1†^, Yujiao Wang^2†^, Jing Zeng^1^, Zhipeng Cao^1^,
Hong Chen^1*^, Yijun Liu^1^, Qinglin Zhang^1*^, Li Su^1,3*^

1 Sino-Britain Centre for Cognition and Ageing Research, Faculty of Psychology, Southwest University, Chongqing, China

2 Department of Education Science, Liupanshui Normal University, Liupanshui, Guizhou, China

3 Department of Psychiatry, University of Cambridge, Cambridge, UK

* To whom correspondence should be addressed. E-mail: james_psych@yeah.net (QZ), ls514@cam.ac.uk (LS), chenhg@swu.edu.cn (HC). Tel & fax: +86 23 6825 2660.

† The first two authors contributed equally to this work.

**Analyses of response times**

The following analyses are based on data from trials of interest, i.e., trials in which a subject refused to gamble in the high loss condition, chose to gamble in the low loss condition, or refused or chose to gamble in the medium loss condition.

**1. Are the response times later or earlier than the time window of ERP?**

To answer this question with more details, we list the percentiles of response times in all trials of interest, as presented in Table S1. From the table it is clear that in most trials response times are more than 630 ms, i.e., later than the LPC time window in consideration. These long response times suggest the following points. First, the subjects did the task seriously. Second, subjects probably made a tradeoff between the specific gain and loss rather than made the decision simply according to their fear about risk. The former should require more time than the latter.

|  | Ratio of loss/gain | | |
| --- | --- | --- | --- |
| Percentiles | 0.1-0.2 | 0.5-0.6 | 0.9-1.0 |
| 5 | 583 | 507 | 497 |
| 10 | 628 | 579 | 564 |
| 15 | 659 | 620 | 613 |
| 20 | 684 | 657 | 658 |
| 25 | 709 | 689 | 694 |
| 30 | 738 | 723 | 731 |
| 35 | 763 | 753 | 761 |
| 40 | 785 | 793 | 794 |
| 45 | 811 | 821 | 824 |
| 50 | 841 | 855 | 858 |
| 55 | 874 | 896 | 891 |
| 60 | 910 | 936 | 933 |
| 65 | 946 | 988 | 975 |
| 70 | 994 | 1048 | 1023 |
| 75 | 1047 | 1116 | 1084 |
| 80 | 1112 | 1188 | 1173 |
| 85 | 1183 | 1281 | 1265 |
| 90 | 1322 | 1408 | 1397 |
| 95 | 1546 | 1637 | 1662 |
| **Table S1. Percentiles of response times (ms).** | | | |

**2. Are response times same or different among three conditions?**

To compare the difference in response times among different conditions, we firstly calculated the average response time for each subject by each ratio of loss/gain. Figure S1 presents the descriptive statistics. We then performed a repeated measurement analysis of variance, with dependent variable being response time, independent variable being ratio of loss/gain. *F* (2, 38) = .024; *p* = .929. That is, the response time among the three conditions are intrinsically the same. This situation makes the comparison between low loss condition and high loss condition be more reasonable. This result also suggests that the mental processes were probably the same across all the three conditions and thus using ERPs in two conditions to predict behavior in the 3^rd^ condition is feasible.

**Figure S1. The average response time for each condition.**


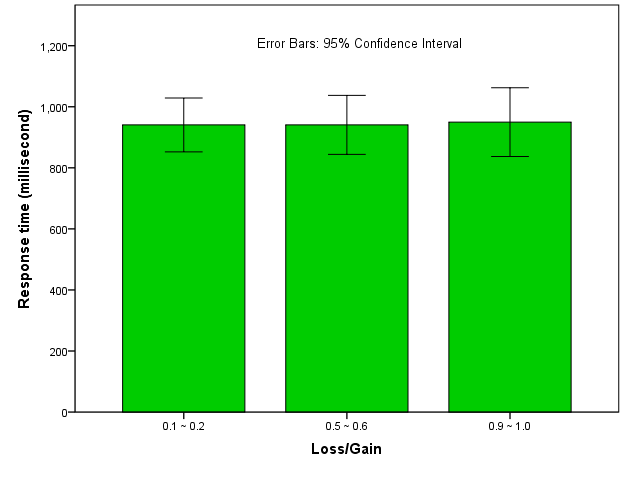

Supplement: Supplementary file 1 — Supplementary Information [file 41598_2019_41242_MOESM1_ESM.docx]
